# Discharge outcomes among elderly patients undergoing emergency abdominal surgery: registry study of discharge data from Irish public hospitals

**DOI:** 10.1186/s12877-020-1469-4

**Published:** 2020-02-19

**Authors:** Aisling McCann, Jan Sorensen, Deirdre Nally, Dara Kavanagh, Deborah A. McNamara

**Affiliations:** 1 National Clinical Programme in Surgery, Royal College of Surgeons in Ireland, 2 Proud’s Lane, Dublin, Ireland; 2 Healthcare Outcomes Research Centre, Royal College of Surgeons in Ireland, Beaux Lane House, Mercer Street Lower, Dublin, Ireland; 3 Department of Surgical Affairs, Royal College of Surgeons in Ireland, 121 St. Stephen’s Green, Dublin, Ireland

**Keywords:** Emergency intra-abdominal surgery, Length of stay, Nursing home capacity, Ireland

## Abstract

**Background:**

Intra-abdominal emergency surgery is associated with high mortality risk and long length of hospital stay. The objective of this study was to explore variations in surgery rates, the relationship between admission source and discharge destination, and whether the postoperative length of stay was related to nursing home capacity in Irish counties.

**Methods:**

Data on emergency hospital episodes for 2014–18 for patients aged over 65 years with a primary abdominal procedure code were obtained from the National Quality Assurance Improvement System. Data on population and nursing home capacity were obtained from the Central Statistics Office and the Health Information and Quality Authority. Episode rates per 100,000 were estimated for sex and age groups and compared between 26 Irish counties. The association between admission source and discharge destination was explored in terms episode numbers, length of stay and mortality. A negative binomial regression model estimated casemix adjusted excess post-operative length of stay. The correlation between excess post-operative length of stay and nursing home capacity was explored by linear regression.

**Results:**

Overall, 4951 hospital episodes were included. The annual surgery rate ranged from 100 episodes per 100,000 65–69 years old to 250 per 100,000 85–89 year old men. 90% of the episodes were admitted from patients’ home. Four in five of these patients returned to their home while 12.7% died at hospital. The proportion of episodes where patients returned to their home reduced to two in five for those aged 85–89 years. The post-operative length of stay was 13.6 days longer (*p* < 0.01) for episodes admitted from home and discharged to nursing home in comparison with episodes discharged home. A negative association (*p* = 0.08) was found between excess post-operative length of stay and county-level nursing home capacity.

**Conclusions:**

This study provides relevant information to support informed consent to surgery for patients and clinicians and to improve the provision of care to older patients presenting with intra-abdominal emergencies.

## Background

Older patients who undergo intra-abdominal surgery are often frail and have complicating underlying medical comorbidities [1–4] which mean these operations are associated with significant post-operative morbidity and mortality [5]. In contrast to planned surgery, there is less time to prepare emergency patients preoperatively, although such preparations would be valuable in this patient group [6]. As a consequence, older patients are vulnerable to post-operative functional decline that can have an impact upon quality of life [7]. Temporary or permanent changes in their care needs after surgery may have profound implications for patients, family and carers, and society.

The configuration of Irish publically-funded healthcare services, and data available from administrative health databases are conducive for population-level research into emergency intra-abdominal surgery. Previous studies have shown an overall in-hospital mortality of 77 per 1000 emergency episodes and demonstrated a survival advantage for patients treated by high volume surgical teams [8].

An increasing number of elderly patients undergo emergency abdominal surgery which reflects demographic change, longer life expectancy, and greater acceptance of emergency operative management in older people [9]. Evaluations of outcomes from emergency surgery for older patients, and the consent process for such procedures, often focus on the ability to prolong life instead of describing potential impacts on function and ability for independent living [7, 10]. A comprehensive understanding of not just survival, but also of the flow of older patients through hospital services is required to ensure that healthcare systems meet the needs of older people. Older survivors of intra-abdominal procedures may experience reduced independence, have different rehabilitation needs and a greater risk of requiring long-term residential care [2]. Healthcare systems must plan for these aspects to achieve best outcomes and avoid unwanted adverse impacts on provision of other hospital services.

The aim of this study was to investigate the discharge outcomes among elderly patients who were admitted to an acute hospital and received emergency abdominal surgery as the primary procedure during their hospital stay. The primary objective was to determine differences in abdominal surgical rates by sex and age groups nationally and by county of residence. A secondary objective was to analyse patient flow to identify relationships between admission sources and discharge destinations. We also wished to determine the extent to which nursing home capacity at county level influences the discharge rate to this destination and post-surgery length of stay.

## Methods

### Health care context

Publicly funded health care in the Republic of Ireland (population 4.8 million) is available to all residents. It is organized and delivered by the Health Service Executive (HSE). Twenty-four hospitals provide acute surgical services, each operating an emergency on-call rota. The structure of the health services is such that almost all emergency abdominal surgery is performed in public hospitals and information about these episodes of care is collected in a single national database. In each hospital, data from patient medical records is coded by trained coders and submitted to the national Hospital Inpatient Enquiry (HIPE) System. The National Quality Assurance Improvement System (NQAIS) is a data extraction system by which HIPE data can be retrieved and analysed.

Both public and private long-stay care facilities are available in the Republic of Ireland. The HSE provides public nursing homes and private institutions provide private nursing homes. The Health Information and Quality Authority (HIQA) is the government-funded agency responsible for the regulation of healthcare and social care systems. HIQA’s Older People’s Inspection Team is legally responsible for the monitoring, inspection and registration of designated centres for older people, such as nursing homes (https://www.hiqa.ie/), [11]. It is perceived that their registry of nursing home capacity is reasonably accurate as it includes both public and private nursing homes. However, a few smaller private institutions may be missing. Medical cards are provided to citizens from lower socioeconomic groups, those with certain chronic medical conditions and/or greater than 70 years. Holders are entitled to certain health services free of charge.

This national, population-based study using NQAIS data is reported according to STROBE guidelines [12].

### Data extraction and categorisation

Clinical experts defined procedure codes corresponding to emergency abdominal surgery, based on the Australian Classification of Interventions in Health (ACHI). To be comparable with the UK National Emergency Laparotomy Audit (NELA) work appendectomy, cholecystectomy, aortic and trauma surgical emergency procedures were excluded. The full list of included procedures is available as supplementary material in [8]. Data on emergency episodes for individuals aged 65 years or older with these codes were obtained from the NQAIS database for the study period January 1st 2014 - December 31st 2018. The county of residence was coded directly and episodes for patients resident outside the Republic of Ireland were excluded. The data set included the 26 counties.

Episodes were categorised into meaningful variables including five-year age groups, admission source and discharge destination. The pre-score Charlson Co-morbidity Index (CCI) was categorised into 6 groups with increasing scores. Cancer diagnosis was coded using primary ICD-10 diagnostic codes (C17, C18, C19, C78) as a dichotomous variable. Medical card status was recorded to differentiate people less than 70 years of age with and without a medical card. A five-category physical status score based on the American Society of Anaesthesiologists’ (ASA) classification system was missing for 16.8% of the episodes and hence was not included as a descriptive variable (mean score 2.9; SD 0.80; *n* = 4119). Total length of stay (LOS), pre- and post-operative stay and LOS in a critical care unit were coded and used as process outcomes. Readmission after 7 and 30 days were readily available in the data set. Death was identified from the discharge destination, and based on the date of operation and discharge day we could derive dichotomous variables for death within 7 and 30 days after the initial surgical procedure, as well as death at hospital. Information about post-discharge death was not available and currently cannot be linked to the data set.

### Other data sources

Information regarding 2019 nursing home places by county was obtained from HIQA (https://www.hiqa.ie/). Population data from the 2016-census by county, sex and one-year age groups were obtained from the Central Statistical Office (CSO) [13]. Population data were aggregated to five-year age groups and validated with the aggregated number provided by the CSO. Of note, the CSO does not provide similar data for the non-census years. We therefore assumed that the 2016-population data provide a reasonable description of the mean population during the five-year study period.

### Analysis

The analyses were conducted in Stata 16.0. We used the user-written command “basetable” developed by Bruun [14] and Markstat procedures developed by Rodriguez [15]. The “tmap” package for R was used to produce Fig. 3 [16]. *P*-values < 0.05 were considered to indicate statistical significance. The descriptive analysis tested for differences between discharge destinations using chi-squared tests for categorical variables and ANOVA for continuous variables. The national episode rate is presented as the episode rate per 100,000 population aged over 65 years.

### Length of stay related to local nursing home capacity

Mean LOS (Standard deviation (SD)) was cross tabulated for patients who were admitted from home and discharged to home or nursing home. Case mix differences were identified between these two patient groups: significantly more females were discharged to nursing homes; patients discharged to nursing homes were older, more likely to be public patients with medical card over 70 years of age, and have a higher CCI. These case mix differences were adjusted for in a negative binomial regression model of the post-operative LOS (count variable regression). Ordinary least square (OLS) regression provided a poorer fit to the data.

Predictions from the specified model were used to obtain a set of adjusted LOS predictions, and hence calculate the individual difference between the observed and predicted LOS. A measure of additional LOS was then constructed for individuals with observed stay longer than the predicted stay. Patients with shorter observed stay than the predicted stay were assigned a zero value. Excess LOS was calculated as the stay beyond the predicted stay. The relation between the county specific mean excess stay and the rate of nursing homes per 100,000 people aged over 65 years were explored graphically and in a linear model. A negative parameter indicates an inverse relationship between excess stay and nursing home capacity.

### Approval

This study was endorsed within the NQAIS Clinical Governance Framework. Ethical approval was granted by the Research Ethics Committee of the Royal College of Surgeons of Ireland (REC001534). Data on individual hospitals and surgical teams are anonymised to ensure confidentiality. There was no patient or public involvement in this study.

## Results

### National episode rates by sex and age

**Fig. 1:**
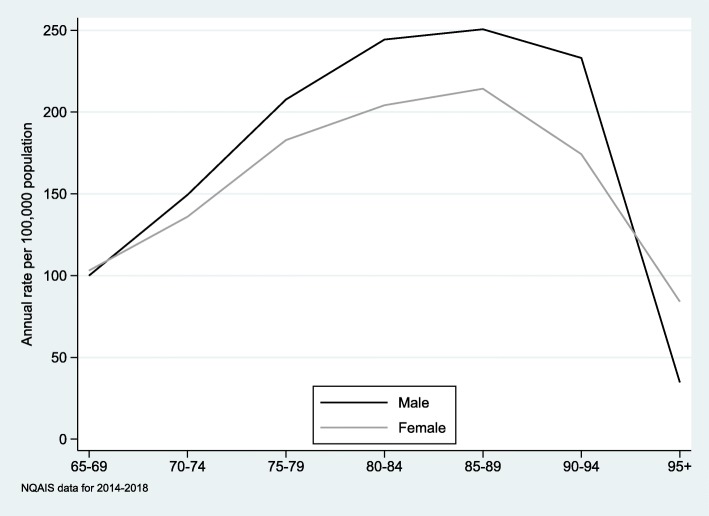
Annual sex and age related rates of episodes with emergency abdominal surgery per 100,000 population

During the years 2014 to 2018 inclusive, emergency abdominal surgery was performed in 4951 episodes for patients aged 65 years or older. The total number of episodes increased slightly each year, although the annual episode rate per 100,000 population remained within the range 148 to 162 per 100,000 over the study period. Table 1 summarises the patient characteristics by sex. There were more female than male patients (*p* = 0.02). Males patients were slightly younger than females (75.6 vs 76.5; *p* < 0.01), the male average Charlson score was higher (7.6 vs 5.8, *p* > 0.01), and more males were admitted with a cancer diagnosis (25.8% vs 19.7%, *p* < 0.01). Figure 1 demonstrates the increasing episode rate associated with age until 90 years, and a significantly higher episode rate for males at all ages between age 65 to 90. The annual episode rate for 65–69 year olds was 100 episodes per 100,000 males or females and peaked for 85–89 years old where the rate for males was 250 per 100,000 and for females 214 per 100,000.

**Table 1:** Descriptive analysis of male and female patients aged over 65 years, who have had emergency abdominal surgery

n (%)	Male	Female	Total	*p*-value
	2383 (48.1)	2568 (51.9)	4951 (100.0)	
Age group, n (%)				
65–69	525 (22.0)	548 (21.3)	1073 (21.7)	
70–74	594 (24.9)	563 (21.9)	1157 (23.4)	
75–79	562 (23.6)	561 (21.8)	1123 (22.7)	
80–84	430 (18.0)	468 (18.2)	898 (18.1)	
85–89	209 (8.8)	302 (11.8)	511 (10.3)	
90+	63 (2.6)	126 (4.9)	189 (3.8)	< 0.01
Admission source, n (%)				
Home	2129 (89.3)	2339 (91.1)	4468 (90.2)	
Other Hospital	165 (6.9)	155 (6.0)	320 (6.5)	
Nursing Homes	89 (3.7)	74 (2.9)	163 (3.3)	0.10
Public or private patient, n (%)				
Public	1707 (71.6)	1778 (69.2)	3485 (70.4)	
Private	676 (28.4)	790 (30.8)	1466 (29.6)	0.07
Medical Card, n (%)				
No Medical Card	681 (28.6)	646 (25.2)	1327 (26.8)	
Medical card u70	292 (12.3)	346 (13.5)	638 (12.9)	
Medical card o70	1410 (59.2)	1576 (61.4)	2986 (60.3)	0.02
Charlson Comorbidity Index, n (%)				
0	1064 (44.6)	1414 (55.1)	2478 (50.1)	
1–3	137 (5.7)	119 (4.6)	256 (5.2)	
4–6	153 (6.4)	170 (6.6)	323 (6.5)	
7–9	166 (7.0)	163 (6.3)	329 (6.6)	
10+	863 (36.2)	702 (27.3)	1565 (31.6)	< 0.01
Has cancer diagnosis, n (%)				
No	1769 (74.2)	2061 (80.3)	3830 (77.4)	
Yes	614 (25.8)	507 (19.7)	1121 (22.6)	< 0.01
LOS total, mean (sd)	27.7 (34.2)	27.5 (35.8)	27.6 (35.0)	0.79
LOS pre-op, mean (sd)	5.8 (13.9)	5.1 (11.8)	5.4 (12.9)	0.08
LOS post-op, mean (sd)	21.9 (29.7)	22.3 (31.7)	22.1 (30.8)	0.66
ICU / CCU bed days, mean (sd)	4.4 (11.1)	3.8 (8.2)	4.1 (9.7)	0.03
Readmission after 7d, mean (sd)	0.04 (0.21)	0.04 (0.20)	0.04 (0.20)	0.58
Readmission after 30d, mean (sd)	0.12 (0.32)	0.11 (0.31)	0.11 (0.32)	0.33
Death 7d post-OP, mean (sd)	0.05 (0.23)	0.06 (0.24)	0.06 (0.24)	0.18
Death 30d post-OP, mean (sd)	0.10 (0.31)	0.11 (0.32)	0.11 (0.31)	0.44

### Patient flow by admission sources and discharge destinations

Table 2 summarises differences in patient characteristics for episodes stratified by discharge destination (discharged to home, nursing home, transferred to other hospitals or died in hospital). 90.2% of the episodes were admitted from home, 6.5% from other hospitals and 3.3% from nursing homes. Of the episodes admitted from patients’ home 60.8% were discharged to home, while 20% were discharged to other hospitals and 7% were discharged to nursing homes and 12.7% died. This compares to 80.6% of episodes admitted from nursing homes were discharged to the nursing home and 18.4% died. 26.3% of episodes for patients admitted from other hospitals were discharged to nursing homes and 14.1% died (Table 3).

**Table 2:** Descriptive analysis by discharge destination for patients aged over 65 years, who have had emergency abdominal surgery

n (%)	Home	Hospital transfer	Nursing Home	Death	Total	*p*-value
	2865 (57.9)	389 (7.9)	1055 (21.3)	642 (13.0)	4951 (100.0)	
Sex, n (%)						
Male	1430 (49.9)	175 (45.0)	473 (44.8)	305 (47.5)	2383 (48.1)	
Female	1435 (50.1)	214 (55.0)	582 (55.2)	337 (52.5)	2568 (51.9)	0.02
Age group, n (%)						
65–69	801 (28.0)	66 (17.0)	130 (12.3)	76 (11.8)	1073 (21.7)	
70–74	781 (27.3)	82 (21.1)	165 (15.6)	129 (20.1)	1157 (23.4)	
75–79	644 (22.5)	97 (24.9)	239 (22.7)	143 (22.3)	1123 (22.7)	
80–84	413 (14.4)	75 (19.3)	270 (25.6)	140 (21.8)	898 (18.1)	
85–90	171 (6.0)	50 (12.9)	183 (17.3)	107 (16.7)	511 (10.3)	
90+	55 (1.9)	19 (4.9)	68 (6.4)	47 (7.3)	189 (3.8)	< 0.01
Admission source, n (%)						
Home	2715 (94.8)	305 (78.4)	881 (83.5)	567 (88.3)	4468 (90.2)	
Other Hospital	148 (5.2)	84 (21.6)	43 (4.1)	45 (7.0)	320 (6.5)	
Nursing Homes	2 (0.1)	0 (0.0)	131 (12.4)	30 (4.7)	163 (3.3)	< 0.01
Public or private patient, n (%)						
Public	1934 (67.5)	253 (65.0)	795 (75.4)	503 (78.3)	3485 (70.4)	
Private	931 (32.5)	136 (35.0)	260 (24.6)	139 (21.7)	1466 (29.6)	< 0.01
Medical Card, n (%)						
No Medical Card	826 (28.8)	146 (37.5)	223 (21.1)	132 (20.6)	1327 (26.8)	
Medical card u70	463 (16.2)	30 (7.7)	95 (9.0)	50 (7.8)	638 (12.9)	
Medical card o70	1576 (55.0)	213 (54.8)	737 (69.9)	460 (71.7)	2986 (60.3)	< 0.01
Charlson Comorbidity Index, n (%)						
0	1650 (57.6)	165 (42.4)	488 (46.3)	175 (27.3)	2478 (50.1)	
1–3	157 (5.5)	22 (5.7)	48 (4.5)	29 (4.5)	256 (5.2)	
4–6	174 (6.1)	27 (6.9)	63 (6.0)	59 (9.2)	323 (6.5)	
7–9	180 (6.3)	27 (6.9)	65 (6.2)	57 (8.9)	329 (6.6)	
10+	704 (24.6)	148 (38.0)	391 (37.1)	322 (50.2)	1565 (31.6)	< 0.01
Has cancer diagnosis, n (%)						
No	2191 (76.5)	312 (80.2)	792 (75.1)	535 (83.3)	3830 (77.4)	
Yes	674 (23.5)	77 (19.8)	263 (24.9)	107 (16.7)	1121 (22.6)	< 0.01
LOS total, mean (sd)	23.6 (29.5)	34.4 (38.9)	36.3 (40.5)	26.8 (41.4)	27.6 (35.0)	< 0.01
LOS pre-op, mean (sd)	4.8 (11.1)	5.8 (12.1)	6.1 (12.3)	6.8 (19.7)	5.4 (12.9)	< 0.01
LOS post-op, mean (sd)	18.7 (25.2)	28.7 (35.2)	30.2 (37.5)	20.0 (34.8)	22.1 (30.8)	< 0.01
ICU / CCU bed days, mean (sd)	2.6 (6.8)	6.9 (13.2)	4.3 (10.1)	8.7 (14.7)	4.1 (9.7)	< 0.01
Readmission after 7d, mean (sd)	0.05 (0.22)	0.03 (0.17)	0.05 (0.22)	0.00 (0.00)	0.04 (0.20)	< 0.01
Readmission after 30d, mean (sd)	0.13 (0.34)	0.09 (0.29)	0.14 (0.35)	0.00 (0.00)	0.11 (0.32)	< 0.01
Death 7d post-OP, mean (sd)	0.00 (0.00)	0.00 (0.00)	0.00 (0.00)	0.45 (0.50)	0.06 (0.24)	< 0.01
Death 30d post-OP, mean (sd)	0.00 (0.00)	0.00 (0.00)	0.00 (0.00)	0.84 (0.37)	0.11 (0.31)	< 0.01

**Table 3:** Place of admission and discharge destination (number of patients, row %)

Admission source	Discharge destination									
	Home		Hospital transfer		Nursing Home		Death		Total	Col %
Home	2715	60.8%	305	6.8%	881	19.7%	567	12.7%	4468	90%
Other Hosp.	148	46.3%	84	26.3%	43	13.4%	45	14.1%	320	6%
Nursing Homes	–		–		133	81.6%	30	18.4%	163	3%
All	2863	57.9%	389	7.9%	1057	21.3%	642	13.0%	4951	100%

Note: Low numbers of patients admitted from nursing home and discharged to either home or hospital transfer have been included as discharged to nursing home to avoid with potential identification

**Fig. 2:**
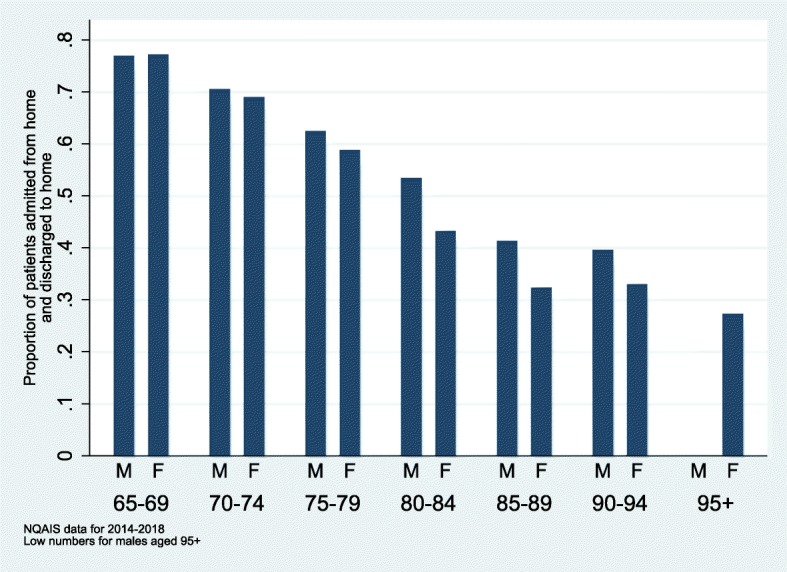
Proportion of patients admitted from home who were discharged to home (*n* = 2715)

Figure 2 shows that the proportion of episodes admitted from home who return home for different sex and age groups. Not surprisingly, the proportions reduce by age and are slightly lower for females in the older age groups. There were little difference in proportions with adjustment for sex, age, Charlson comorbidity index and cancer diagnosis. Among the 85–89 year-old males, 41.3% (95% CI: 34.2%;48.4%) returned to their home whereas for women aged 85–89 years, the proportion was 32.4% (95% CI: 26.8%;37.9%).

### LOS analysis by admission sources and discharge destination

The average LOS and post-operative LOS for patients admitted from home, other hospitals and nursing homes by their discharge destination is shown in Table 4. The average LOS in the total population was 27.5 days (SD 27.6; range 1–613 days). Patients admitted from their home and discharged to their home stayed on average 23.1 days, while those discharged to nursing homes stayed at hospital an additional 15.5 days (*p* < 0.01).

**Table 4:** Total and post-operative length of stay by admission source and discharge destination (mean (sd) [n])

Admission source	Discharge destination				
	Home	Hospital transfer	Nursing Home	Death	All
Total length of stay					
Home	23.1 (27.1) [2715]	35.1 (38.6) [305]	38.6 (42.9) [881]	27.2 (42.2) [567]	27.5 (34.3) [4468]
Other hospitals	32.8 (57.5) [148]	32.1 (40.2) [84]	29.5 (21.6) [43]	27.6 (35.7) [45]	31.4 (46.7) [320]
Nursing homes	–	–	23.8 (22.2) [133]	18.9 (35.0) [30]	22.9 (25.0) [163]
All	23.6 (29.5) [2863]	34.4 (38.9) [389]	36.3 (40.5) [1057]	26.8 (41.4) [642]	27.6 (35.0) [4951]
Post-operative length of stay					
Home	18.3 (24.1) [2715]	29.0 (34.2) [305]	32.2 (39.9) [881]	20.2 (35.2) [567]	22.0 (30.6) [4468]
Other hospitals	25.5 (39.3) [148]	27.5 (38.6) [84]	26.0 (19.8) [43]	22.2 (31.8) [45]	25.6 (35.9) [320]
Nursing homes	–	–	19.3 (19.9) [133]	13.8 (32.0) [30]	18.3 (22.6) [163]
All	18.7 (25.2) [2863]	28.7 (35.2) [389]	30.2 (37.5) [1057]	20.0 (34.8) [642]	22.1 (30.8) [4951]

Note: Low numbers of patients admitted from nursing home and discharged to either home or hospital transfer have been included as discharged to nursing home to avoid with potential identification

Patients who were admitted from a nursing home and discharged to the nursing home stayed an average 23.1 days at hospital.

Patients stayed on average 5.4 days in hospital before they had the surgical procedure. There were only small and non-significant differences between preoperative stay for patients admitted from home, other hospitals and nursing homes (*p* = 0.61).

A significant difference in post-operative LOS was observed between patients admitted from home (22 days), patients admitted from other hospitals (25.6 days) and patients admitted from nursing homes (18.3 days).

Patients who were admitted from home and discharged to nursing homes stayed for 13.9 days more than patients discharged to home (32.2 vs 18.3 days; *p* < 0.01). Patients admitted from home and discharged to nursing home also stayed 13.6 days (*p* < 0.01) longer than patients admitted from nursing home and discharged to a nursing home.

### Nursing home capacity

**Fig. 3:**
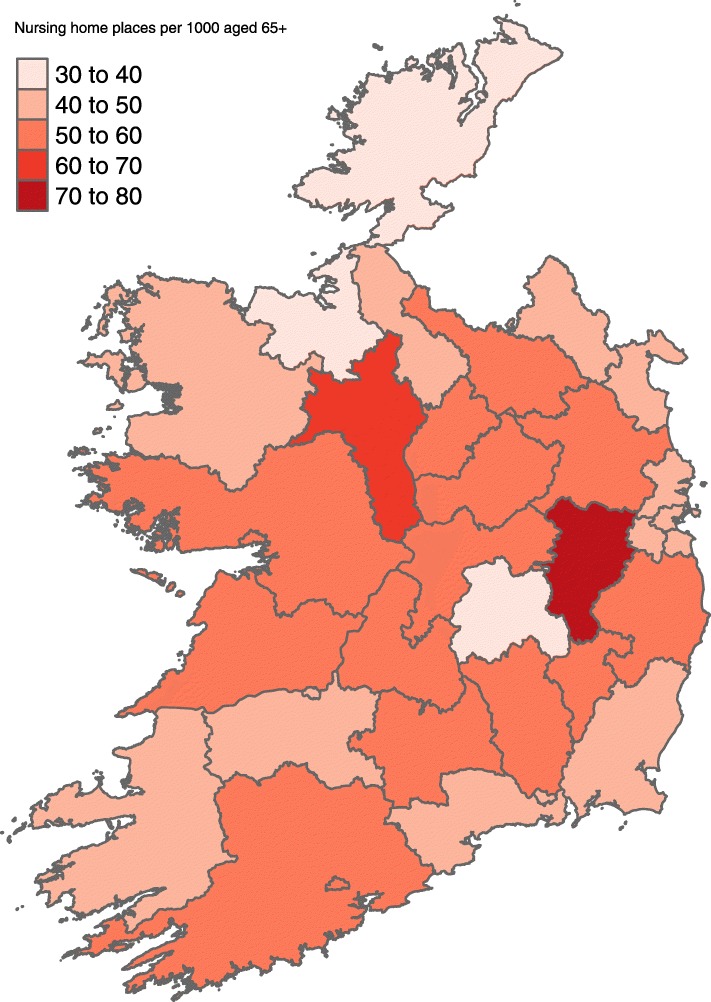
County specific nursing home places per 100,000 population aged over 65 years

At national level there were 49.5 nursing home places per 100,000 population aged over 65 years. Figure 3 shows the variation in nursing home capacity by county. The capacity ranged from 31.4 to 73.7 per 100,000 population.

### Length of stay related to local nursing home capacity

Of the 3596 patients who were admitted from home and discharged either home or to a nursing home, 1041 (28.9%) had longer hospital stay than case-mix adjusted expected stay. There was a statistically significant difference between patients discharged to home and nursing home (24.4% vs 43.0%; OR = 2.3; p < 0.01). There was no statistically significant difference in this proportion across counties although the Odds ratio (OR) varied from 0.44 to 1.74.

**Fig. 4:**
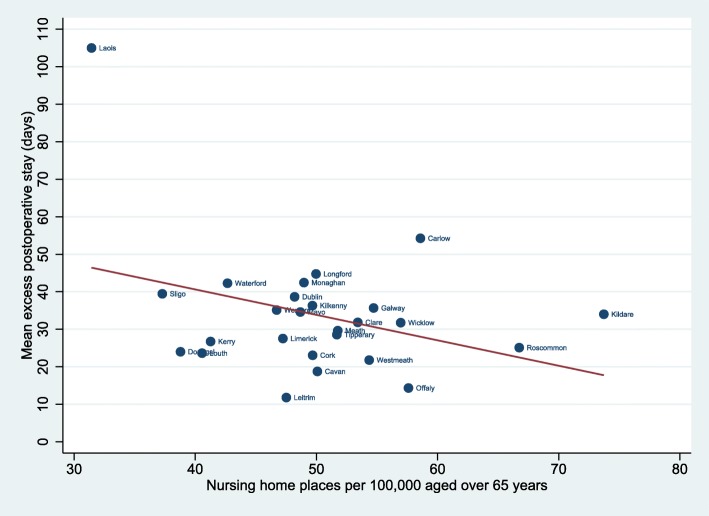
Association between the extended postoperative hospital stay and nursing home capacity per 100.000 people aged 65+ in the different counties

Figure 4 shows the association between the extended post-operative hospital stay and the nursing home capacity per 100,000 population aged over 65 years in the difference counties. The linear regression imposed in Fig. 4 shows a negative correlation (− 0.68; *p* = 0.08) suggesting that the extended post-operative stay is shorter in counties with large nursing home capacity.

## Discussion

This population-level study has quantified outcomes including in-hospital mortality, discharge destination and length of stay for patients aged over 65 years undergoing emergency abdominal surgery in Irish hospitals during 2014–2018. The overall in-hospital mortality rate for patients over 65 was 130 per 1000 and increased to 184 per 1000 for patients over 80 years. A mortality rate of 77 per 1000 episodes in a population aged 16 and over has previously been reported [8]. This elevated mortality compares favourably with the first UK NELA report that demonstrated a mortality rate of 244 per 1000 in patients aged 80 or over [5]. We observe an unexplained variation in the proportion of male and female patients of similar age undergoing the procedure and demonstrate an increased need for post-operative nursing home support in this cohort of older patients.

The findings of this study are important in designing a health system that meets the in-hospital, rehabilitation and placement needs of older patients, and are relevant for assisting patients and their families to better understand the risks associated with this type of surgery including the in-hospital mortality as well as the higher risk of post-operative dependency in surviving patients.

The rates of patients undergoing these procedures increase with age up to the age of 90 years, and increases in morbidity and mortality by age are also observed. Previous research has been conflicting with regards to the influence of age on mortality following emergency surgery. In older reports, chronological age is reported to be an independent risk factor for mortality, with mortality reported to increase by approximately 4% for each 10 years of additional age [5]. More recent studies, adjusting for pre-operative morbidity as captured by the ASA classification system and various frailty scores have found that increasing morbidity and frailty, as opposed to simply chronological age, are the independent risk factors [17, 18]. For example, in a series of 220 patients, higher frailty index was associated with in-hospital complications (OR = 2.13; 95% CI; 1.09–4.16; *p* = 0.02) and major complications (OR = 3.87; 95% CI: 1.69–8.84; *p* < 0.01) [17]. Although frailty measures do not replace clinical judgement in the assessment of patients who will benefit from surgical intervention, they may provide a valuable support in decision-making.

The rate of emergency abdominal surgery among men aged over 65 years is higher than among women (Fig. 1). This variation cannot be explained by variables in this dataset although gender differences in the presentation of conditions including colorectal cancer have previously been identified [19]. Late presentation of conditions that could otherwise be treated electively might contribute to the observed difference. Previous research has shown that women have better outcomes after abdominal surgery [4, 20], suggesting the incidence of salvage surgery may be higher in men. Differences in rates of intervention by gender have been previously identified; men are 1.8 times more likely to undergo surgery for an intact abnormal aortic aneurysm than women [21] and 3.3 times more likely to be offered cardiac surgery [22]. Importantly, our dataset does not enable identification of patients who were considered for, but who refused or were denied surgery. Gender differences were also present in this cohort with male patients being older, having a higher CCI and more frequently a cancer diagnosis, although no statistically significant gender difference was found in admission source and length of stay. Female patients were more likely to be discharged to nursing home (22.7% vs 19.9%; *p* = 0.02).

The source of admission is highly influential on the discharge destination. Patients aged over 65 who require emergency surgery and were admitted from a nursing home had more than an 80% incidence of also being discharged to a nursing home and an in-hospital mortality of 18%, compared to rates of 20 and 13% respectively for patients admitted to hospital from home. The initial reason for admission to the nursing home is also relevant. For example, pre-existing dementia influences surgical outcomes: patients with dementia have a higher mortality rate than patients without cognitive impairment [23].

In addition to obvious fiscal consequences, discharge to a nursing home suggests a significant deterioration in levels of independence and, likely, quality of life after emergency abdominal surgery. Validated assessments of quality of life changes after these procedures are challenging due to the emergency nature of their presentation. The rapid deterioration in clinical conditions makes it difficult to make baseline assessment without bias related to recall and non-response.

For patients admitted from home or from another hospital, discharge to a nursing home (19.3%) was a more common outcome than mortality (12.8%) and may be important for patients and their families although current consent practices focus primarily on mortality risk. This would imply that the odds for not being discharged to nursing home or death is 4.2 for patients aged 65–69, reducing to 1 (i.e. a 50dl:50dl chance of returning home) for the over-80 age group. For that reason, morbidity and the ability to live at home are important factors for patients and their family members to consider prior to undergoing emergency surgery. Discharge to a nursing home may happen for a number of reasons: it may be the patient’s usual residence, it may be needed for a short or long period of rehabilitation, or the patient may have become incapable of independent living as a result of their illness. The excess post-operative LOS associated with nursing home discharge (33.8 vs 22.7 days) may reflect more severe illnesses among patients discharged to nursing homes or could indicate that patients are fit to leave hospital but have no access to the step-down nursing home place that they require. Relatively long post-operative hospitalization is observed in our population, a factor that has been associated with reduced function [24]. The UK NELA reports a median post-operative LOS of 11 days but lacks description of discharge destination and includes all ages [5]. In contrast, our study which included only patients older than 65 years observed a mean post-operative LOS of 22 days. Patients who were admitted from home and discharged to a nursing home had a LOS 15.5 days longer than patients discharged home. There was a relationship between longer post-operative LOS and lower nursing home capacity in the patient’s county of residence, although this was not significant (*p* = 0.08). This may suggest some of the prolongation in LOS results from delay in discharge to nursing home facilities.

There are a number of limitations to this study. NQAIS is based on an administrative dataset and hospital episodes were extracted using primary procedure codes so the quality of clinical record keeping and the accuracy of coding performed at each hospital site is a key limitation. There is no unique patient identifier so prior procedures and patients transferred to other hospitals and having subsequent complications are not included in the dataset. A further limitation in this cohort is the lack of a frailty score in the dataset. The nursing home data was retrieved from the HIQA website, which is responsible for the regulation of all nursing homes in the Republic of Ireland, however it may be missing some smaller private entities. Despite these limitations, this dataset includes every patient treated in a publically funded hospital over the 5-year period, representing the single largest dataset on this population currently available.

## Conclusion

The rate of patients undergoing emergency abdominal surgery increases with age, as does mortality. Patients aged 65–69 years undergoing emergency abdominal surgery are 4 times more likely to be discharged home or to another hospital than to a nursing home or to die but This reduces to a 50dl:50dl chance for patients over 80. Patients discharged to a nursing home experience longer LOS. This may partly be explained by nursing home capacity in the patient’s county of residence. This study provides important information to support a better process of informed consent for patients undergoing emergency abdominal surgery and help to improve the planning for a system of care that better meets the needs of older patients who present with an intra-abdominal emergency.

## Data Availability

The data applied in this study are available from NQAIS but restrictions apply to the availability of these data, which were used under license for the current study, and so are not publicly available. Data used and analysed during the current study are available from the corresponding author on reasonable request subject to permission from NQAIS.

## Abbreviations

ACHI: Australian Classification of Interventions in Health
ASA: American Society of Anaesthesiologist
CCI: Charlson Co-morbidity Index
CSO: Central Statistical Office
HIPE: Hospital Inpatient Enquiry
HIQA: Health Information and Quality Authority
HSE: Health Service Executive
LOS: Length of stay
NELA: National Emergency Laparotomy Audit
NQAIS: National Quality Assurance Improvement System
OLS: Ordinary least square
OR: Odds ratio
SD: Standard deviation
